# Retrospective analysis of real‐world prescribing data for managing cisplatin‐based chemotherapy‐induced nausea and vomiting in China

**DOI:** 10.1002/cam4.7121

**Published:** 2024-03-21

**Authors:** Xia Si, Hongyan Zhang, Qingming Ding, Gang Liu, Lin Huang, Xin Sun

**Affiliations:** ^1^ Department of Pharmacy Peking University People's Hospital Beijing China; ^2^ Orthopedic Oncology Peking University People's Hospital Beijing China

**Keywords:** antiemetic, cisplatin‐based chemotherapy, nausea, prescribing, vomiting

## Abstract

**Background:**

The current utilization of neurokinin‐1 receptor antagonists (NK1RAs) and the impact of updated guidelines on prescription patterns of antiemetic drugs among Chinese patients receiving highly emetogenic chemotherapy (HEC) remain undetermined. This study aims to analyze the present situation of Chinese cancer patients using antiemetic drugs and assess the appropriateness of antiemetic regimens.

**Methods:**

Prescription data were collected between January 2015 and December 2020 from cancer patients receiving cisplatin‐based chemotherapy at 76 hospitals in six major cities in China. Trends in the use of antiemetic drugs, prescribing patterns and adherence to antiemetic guidelines were assessed.

**Results:**

Among the 108,611 patients included in this study, 6 classes and 17 antiemetic drugs were identified as monotherapy or combination therapy in 93,872 patients (86.4%), whereas 14,739 patients (13.6%) were administered no antiemetic treatment. 5‐hydroxytryptamine 3 receptor antagonists (5‐HT_3_RAs) and glucocorticoids were the two most frequently used classes of antiemetics, followed by metoclopramide. NK1RAs were underused across the six cities, only 9332 (8.6%) and 1655 (1.5%) cisplatin‐based treatments were prescribed aprepitant and fosaprepitant, respectively. Prescriptions of olanzapine and lorazepam were very low throughout the study period. In prescribing patterns of antiemetic drugs, dual combination regimens were the most common (40.0%), followed by triple combination therapy and monotherapy (25.8% and 15.1%, respectively). Overall, the adherence to antiemetic guidelines for patients undergoing cisplatin‐based regimens was only 8.1% due to inadequate prescription of antiemetic drugs. Finally, our study also revealed that 5‐HT_3_RAs and glucocorticoids were overprescribed in 8.8% and 1.6% of patients, respectively.

**Conclusions:**

The current study reveals suboptimal utilization of recommended antiemetic drugs for managing cisplatin‐based HEC‐induced nausea and vomiting in China. Improving the management of CINV is crucial, and these findings provide valuable insights into optimizing antiemetic drug practices.

## Graphical abstract

The results of our study indicate low adherence to antiemetic guidelines in real‐world clinical practice in China. Only 8.1% of cisplatin‐based chemotherapy received the guideline compliant NK1RA‐5‐HT_3_RA‐glucocorticoid with or without olanzapine therapy when assessing antiemetic drugs administered, which may further reduce when evaluating dose, frequency, and duration. These findings highlight the importance of improving the management of CINV in China and provide useful information for better drug management.

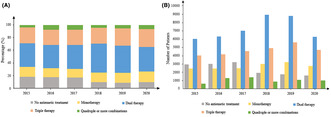

## 1 INTRODUCTION

Despite significant progress in the study and application of new antiemetic drugs, as well as the formulation of evidence‐based guidelines for managing chemotherapy‐induced nausea and vomiting (CINV), lots of patients undergoing chemotherapy are still persistently affected by this dreadful adverse effect.^1^, ^2^ Without appropriate antiemetic prophylaxis, more than 90% of patients undergoing highly emetogenic chemotherapy (HEC) and 30%–90% of those undergoing moderately emetogenic chemotherapy (MEC) are likely to develop nausea and vomiting.^3^ Uncontrolled CINV can cause electrolyte disturbances, dehydration, malnutrition, and esophageal injury,^4^ thereby potentially reducing patients' quality of life,^5^, ^6^ as well as healthcare costs.^7^, ^8^ Severe symptoms can also decrease the adherence of patients and reduce chemotherapy dose, which may further affect anticancer treatment efficacy.^9^

High‐quality evidence‐based antiemetic guidelines have been issued by prominent organizations, such as the National Comprehensive Cancer Network (NCCN), the American Society of Clinical Oncology (ASCO), the Multinational Association of Supportive Care in Cancer/European Society for Medical Oncology (MASCC/ESMO),^3^ the Committee of Rehabilitation and Palliative Care (CRPC) of the Chinese Anti‐Cancer Association, and the Anti‐tumor Drugs Safety Management Committee (ASMC) of the Chinese Society of Clinical Oncology (CSCO).^10^ These widely accessible guidelines consistently recommend tailoring antiemetic prophylaxis to the emetic risk of specific chemotherapy regimens. Studies have shown that patients receiving treatments in accordance with these antiemetic guidelines typically experience improved clinical, economic, and humanistic outcomes.^4^, ^7^, ^11^ Conversely, low adherence to these guidelines in clinical practice results in suboptimal CINV management.^12^, ^13^ In five European countries, only 15% of all HEC and carboplatin‐based treatments adhered to the antiemetic guidelines.^14^ Similarly, large‐scale studies in Asia‐Pacific countries^15^ and the US community^16^ have also reported comparable findings.

In China, cancer is a primary public health problem with heavy disease burden. Effective management of CINV is important as it can potentially improve patient outcomes and reduce healthcare utilization and costs. The overarching goal of CINV control is prevention. In the treatment of HEC, the standard antiemetic regimen comprises a triplet regimen, consisting of single doses of a neurokinin‐1 receptor antagonist (NK1RA), a 5‐hydroxytryptamine 3 receptor antagonist (5‐HT_3_RA), and a glucocorticoid. This regimen forms the foundation for an augmented quadruplet regimen, which includes the atypical antipsychotic drug olanzapine.^3^, ^17^, ^18^ However, NK1RAs were not introduced in China until 2013, and the real‐world patterns of antiemetic drug use, particularly NK1RAs among Chinese patients receiving HEC, have not been thoroughly assessed.^19^ Cisplatin is widely recognized as a highly emetogenic chemotherapeutic agent.^13^ The administration of appropriate antiemetic treatment is critical in preserving the quality of life for patients subjected to HEC. This study utilized a comprehensive clinical prescription database to examine pharmacological treatment strategies for managing CINV associated with cisplatin‐based HEC regimens. Additionally, the study sought to evaluate adherence to antiemetic guidelines in China from 2015 to 2020.

## 2 MATERIALS AND METHODS

### 2.1 Study design and data source

This is a retrospective, open‐label study that examines prescription information from multiple national centers. The data were obtained from the Hospital Prescription Analysis Cooperative Project (HPACP), which is endorsed by the Chinese Pharmaceutical Association. The HPACP database, established in 1997, regularly collects prescription data from participating hospitals on 10 randomly working days each quarter.^20^

### 2.2 Prescription inclusion and data collection

Prescription data of adult cancer inpatients (≥18 years old) receiving cisplatin‐based chemotherapy from January 2015 to December 2020 were extracted. Prescription details, including time, city, hospital code, hospital grade, hospital status (inpatient or outpatient), prescription code, department name, drug code, drug commodity name, drug generic name, route of administration, frequency of administration, single dosage, usage, sex, age, and diagnosis, were obtained from the HPACP database. Data were anonymized and prescription code was used to identify patient identity. Prescriptions with incomplete information were removed. The study received approval from the Ethics Committee of Peking University People's Hospital, with the approval number 2021PHB350‐001. The requirement for written informed consent was waived because the data were analyzed anonymously.

### 2.3 Antiemetic drug exposure

Antiemetic guidelines recommend several antiemetic drugs for prophylaxis in HEC, such as glucocorticoids, 5‐HT_3_RAs, NK1RAs, and olanzapine. Additionally, other classes of drugs, including dopamine antagonists, cannabinoids, and benzodiazepines, may provide additional protective benefits.^21^ To comprehensively assess antiemetic drugs for CINV treatment, our study included six principal pharmacologic classes of antiemetic agents available in China: NK1RAs (aprepitant, fosapitant, netupitant), glucocorticoids (typically dexamethasone), 5‐HT_3_RAs (granisetron, ondansetron, tropisetron, palonosetron, dolasetron, ramosetron, azasetron), atypical antipsychotic (olanzapine), dopamine receptor antagonist (metoclopramide), and benzodiazepine (lorazepam). The study also included NEPA, a fixed‐dose oral combination of the NK1RA netupitant, and the 5‐HT_3_RA palonosetron. Prescription patterns were analyzed in terms of monotherapy and combination therapy. Monotherapy refers to the prescription of only one antiemetic drug on a single prescription, while combination therapy involves the prescription of two or more antiemetic drugs concurrently.

### 2.4 Outcomes and definitions

The study aimed to assess trends in the use of antiemetic drugs, prescribing patterns, and antiemetic‐only guideline adherence (AGA) among patients undergoing cisplatin‐based chemotherapy. AGA was defined as prescription of the appropriate antiemetic agent(s) or class, regardless of the specific dosage or frequency.^22^ The percentage of patients receiving guideline‐compliant therapy was calculated as an indicator of AGA.

### 2.5 Statistical analysis

The study assessed the overall trends and the usage of each antiemetic drug over a 6‐year period. Prescribing patterns for both monotherapy and combination therapy were analyzed. Descriptive statistics were used to analyze the patient demographic characteristics and prescription information. Classification and analyses of data were conducted using Excel 2019 and SPSS version 25.0 (SPSS Inc. Chicago, IL, USA).

## 3 RESULTS

### 3.1 Patient characteristics

A total of 108,611 prescriptions were extracted from 67 tertiary hospitals and 9 secondary hospitals in six principal cities of China (namely Beijing, Shanghai, Tianjin, Guangzhou, Hangzhou, and Chengdu). These cities are spread throughout the northern, eastern, southern, and southwestern areas, representing the most economically developed regions in their respective localities within China. The demographic and clinical characteristics of the patients are delineated in Table 1. Of these patients, 106,864 (98.4%) came from tertiary hospitals and 1747 (1.6%) came from secondary hospitals. The median age of cohort was 56.0 years (ranging from 18 to 120 years), with males accounting for 62.5%. Lung cancer, nasopharyngeal carcinoma, and cervical cancer were identified as the three most prevalent malignant tumors among the study population.

**TABLE 1:** Demographic and clinical characteristics of the patients (*n* = 108,611) receiving cisplatin‐based chemotherapy from 2015 to 2020.

Characteristics	Year					
	2015 (*N* = 16,108)	2016 (*N* = 17,244)	2017 (*N* = 18,728)	2018 (*N* = 19,703)	2019 (*N* = 20,451)	2020 (*N* = 16,377)
Median age (range), years	56 (18–91)	56 (18–96)	55 (18–91)	56 (18–95)	56 (18–95)	56 (18–120)
Age group (years), *n* (%)						
18–29	534 (3.3)	623 (3.6)	646 (3.4)	698 (3.5)	768 (3.8)	591 (3.6)
30–39	1099 (6.8)	1228 (7.1)	1365 (7.3)	1535 (7.8)	1779 (8.7)	1479 (9.0)
40–49	3221 (20.0)	3364 (19.5)	3606 (19.3)	3636 (18.5)	3716 (18.2)	2766 (16.9)
50–59	5281 (32.8)	5476 (31.8)	5893 (31.5)	5935 (30.1)	6254 (30.6)	5149 (31.4)
60–69	4962 (30.8)	5365 (31.1)	5882 (31.4)	6325 (32.1)	6202 (30.3)	4809 (29.4)
70–79	959 (6.0)	1089 (6.3)	1234 (6.6)	1489 (7.6)	1621 (7.926)	1457 (8.9)
≥80	52 (0.3)	99 (0.6)	102 (0.5)	85 (0.4)	111 (0.5)	126 (0.8)
Gender, *n* (%)						
Male	10,106 (62.7)	10,922 (63.3)	11,727 (62.6)	12,266 (62.2)	12,722 (62.2)	10,163 (62.1)
Female	6002 (37.3)	6322 (36.7)	7001 (37.4)	7437 (37.8)	7729 (37.8)	6214 (37.9)
City, *n* (%)						
Beijing	330 (2.0)	293 (1.7)	269 (1.4)	1787 (9.1)	1870 (9.1)	1364 (8.3)
Chengdu	2989 (18.6)	3388 (19.6)	3737 (20.0)	3247 (16.5)	3148 (15.4)	2902 (17.7)
Guangzhou	3986 (24.7)	4487 (26.0)	5287 (28.2)	5822 (29.5)	6535 (32.0)	4703 (28.7)
Shanghai	3807 (23.6)	4262 (24.7)	3945 (21.1)	4204 (21.3)	4053 (19.8)	3435 (21.0)
Tianjin	34 (0.2)	47 (0.3)	534 (2.9)	495 (2.5)	510 (2.5)	466 (2.8)
Hangzhou	4962 (38.8)	4767 (27.6)	4956 (26.5)	4148 (21.1)	4335 (21.2)	3507 (21.4)
Hospital level, *n* (%)						
Tertiary	15,850 (98.4)	17,031 (98.8)	18,495 (98.8)	19,378 (98.4)	20,116 (98.4)	15,994 (97.7)
Secondary	258 (1.6)	213 (1.2)	233 (1.2)	325 (1.6)	335 (1.6)	383 (2.3)
Cancer type (diagnosis), *n* (%)						
Lung cancer	5699 (35.4)	5631 (32.7)	5668 (30.3)	5757 (29.2)	5125 (25.1)	3930 (24.0)
Nasopharyngeal carcinoma	2242 (13.9)	2516 (14.6)	3290 (17.6)	3422 (17.4)	4260 (20.8)	3866 (23.6)
Cervical cancer	1419 (8.8)	1310 (7.6)	1672 (8.9)	1996 (10.1)	2011 (9.8)	1474 (9.0)
Esophagus cancer	905 (5.6)	814 (4.7)	984 (5.3)	1291 (6.6)	1189 (5.8)	737 (4.5)
Ovarian cancer	522 (3.2)	481 (2.8)	615 (3.3)	524 (2.7)	512 (2.5)	424 (2.6)
Breast cancer	374 (2.3)	359 (2.1)	441 (2.4)	478 (2.4)	459 (2.2)	304 (1.9)
Gastric cancer	385 (2.4)	368 (2.1)	392 (2.1)	372 (1.9)	450 (2.2)	363 (2.2)
Malignant lymphoma	311 (1.9)	377 (2.2)	294 (1.6)	349 (1.8)	307 (1.5)	217 (1.3)
Liver cancer	222 (1.4)	215 (1.2)	243 (1.3)	368 (1.9)	279 (1.4)	223 (1.4)
Bladder cancer	153 (0.9)	153 (0.9)	170 (0.9)	256 (1.3)	340 (1.7)	243 (1.5)
Other and unspecified cancers	3876 (24.1)	5020 (29.1)	4959 (26.5)	4890 (24.8)	5519 (27.0)	4596 (28.1)

### 3.2 Overall trends in antiemetic drugs

**FIGURE 1:**
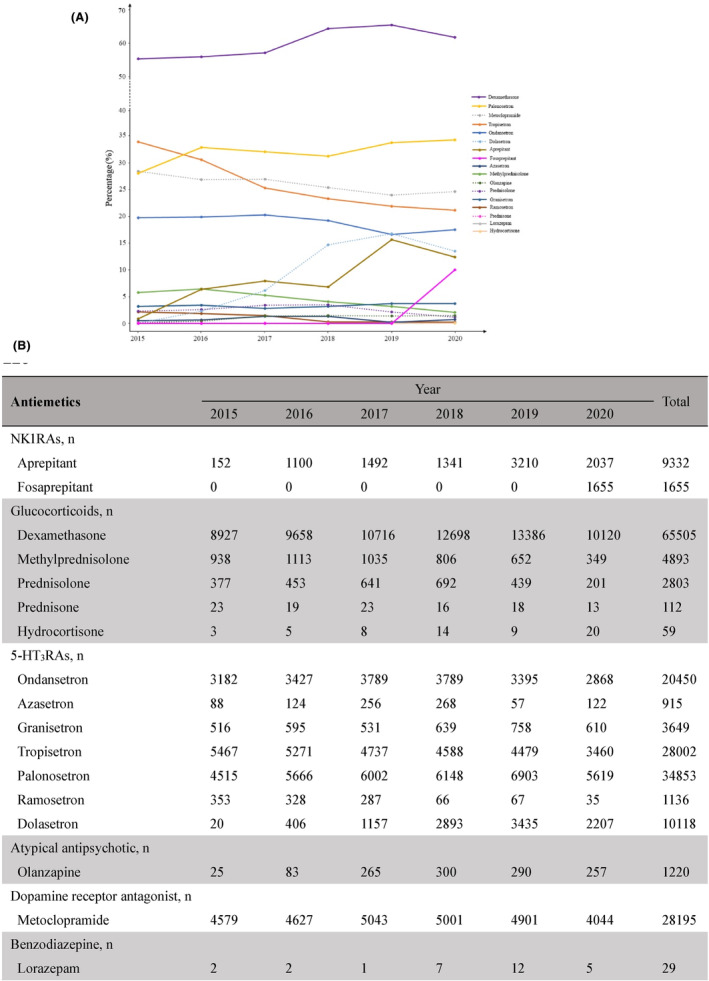
The percentages (A) and counts (B) of patients with each prescribed antiemetic drug from 2015 to 2020 based on each year.

In this study, 6 classes and 17 commonly used antiemetic drugs were identified as monotherapy or combination therapy in the population. The counts and percentages of patients prescribed each class of antiemetic drugs from 2015 to 2020 are presented in Figure 1. 5‐HT_3_RAs were the most frequently prescribed class of antiemetic drugs over the past 6 years, and 89,264 patients (82.2%) receiving at least one 5‐HT_3_RA. Seven 5‐HT_3_RAs, namely ondansetron, tropisetron, palonosetron, dolasetron, granisetron, ramosetron, and azasetron, were used in this retrospective study. In terms of prescription numbers, a second‐generation 5‐HT_3_RA palonosetron was favored over first‐generation 5‐HT_3_RAs throughout this period. As shown in Figure 1, the top three 5‐HT_3_RAs used were palonosetron, tropisetron, and ondansetron. Beginning in 2015, the use of palonosetron gradually increased from 28.0% to 34.3%, while those of tropisetron and ondansetron gradually decreased from 33.94% to 21.1% and from 19.8% to 17.5%, respectively. Dexamethasone, the most commonly used glucocorticoid for preventing CINV, was prescribed to 65,505 patients (60.3%). Other glucocorticoids were utilized in a few patients, including methylprednisolone, prednisolone, prednisone, and hydrocortisone. Two NK1RAs, aprepitant and fosaprepitant, were used during the evaluated year, with aprepitant and fosaprepitant being prescribed in only 9332 (8.6%) and 1655 (1.5%) of cisplatin‐based treatments, respectively. The uses of NK1RAs increased gradually over the years, and this decline in aprepitant use coincided with a significant increase in fosaprepitant prescriptions after its introduction in 2020. Although olanzapine is a viable antiemetic drug for HEC regimens, it was administered to only 1.1% of patients. Despite not being a first‐line recommendation for HEC, metoclopramide was administered to over a quarter of the patients.

### 3.3 No antiemetic treatment

**FIGURE 2:**
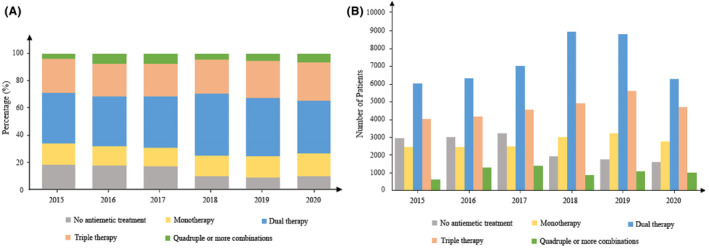
The percentages (A) and numbers (B) of different antiemetic patterns.

Among all cisplatin‐based treatments, 14,445 (13.3%) did not receive any antiemetic treatment for CINV management. Figure 2 illustrates that since 2018, the percentage of cisplatin‐treated patients who did not receive any antiemetic drug has dropped significantly, from over 17% before 2017 to below 10% after 2018.

### 3.4 Prescription patterns of antiemetic drugs

**FIGURE 3:**
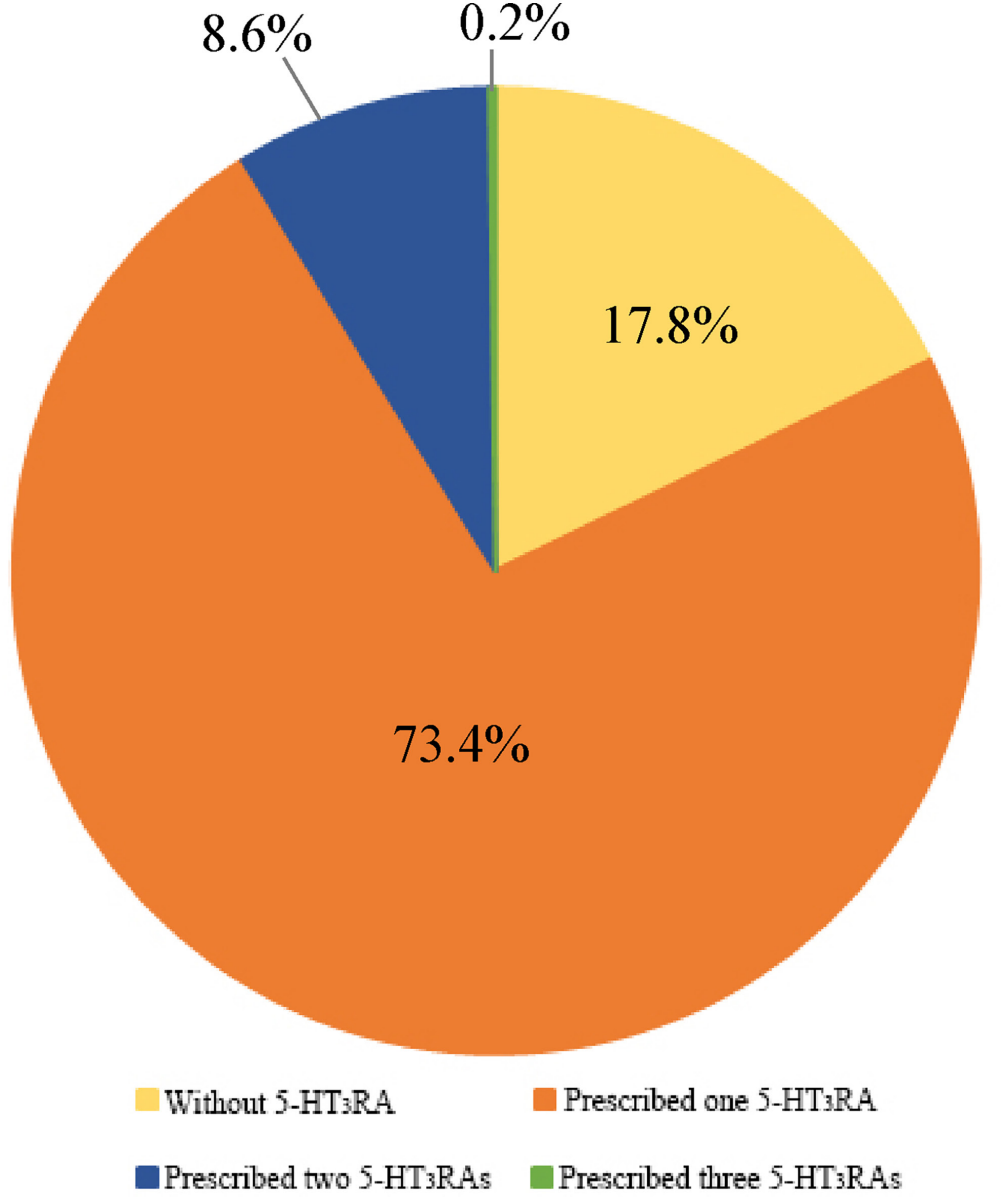
The distribution and proportion of patients who received 5‐HT_3_RAs.

**FIGURE 4:**
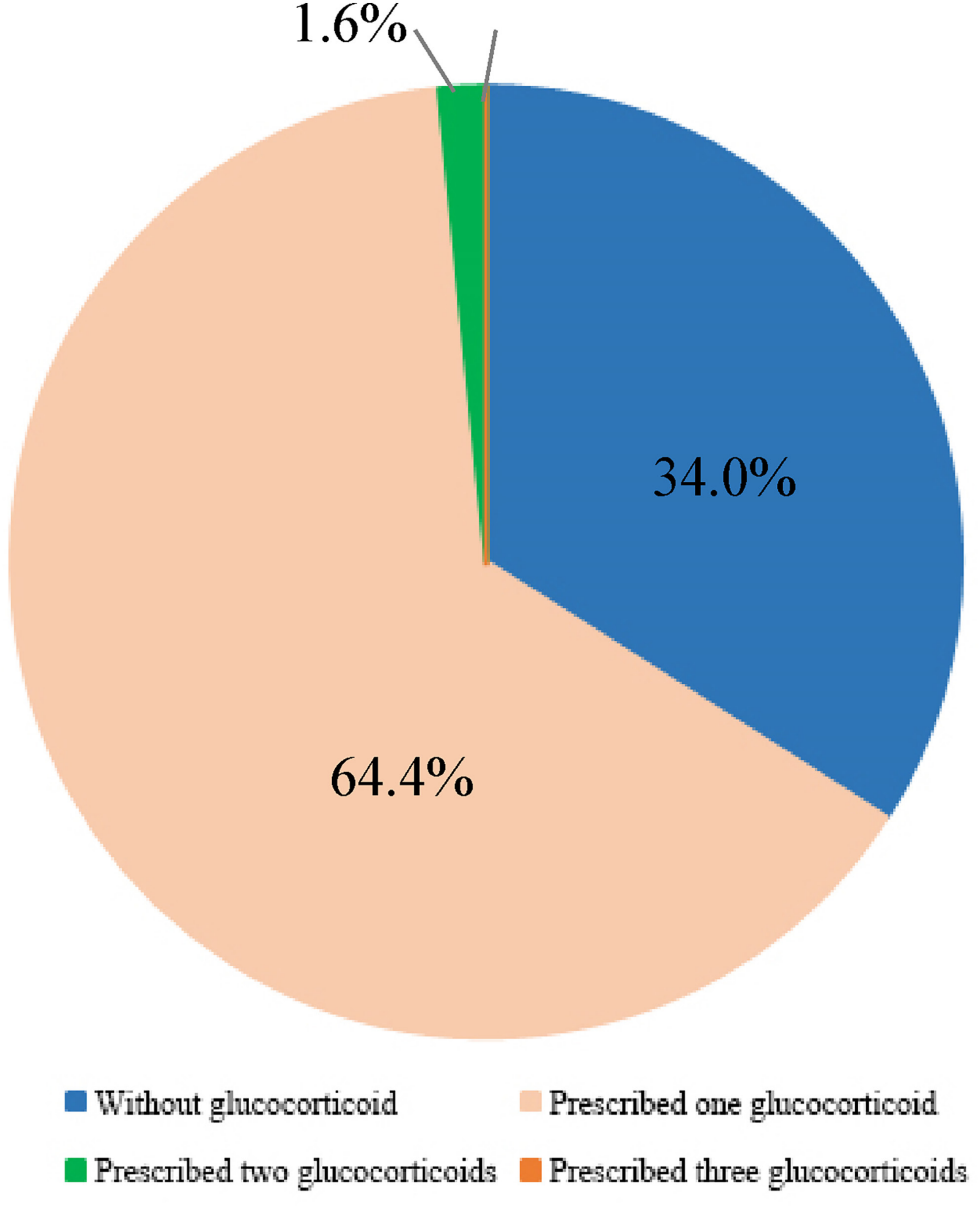
The distribution and proportion of patients who received glucocorticoids.

A total of 94,166 patients (86.7%) received antiemetic treatment during the whole phases of cisplatin‐induced nausea and vomiting (Table 2). Of them, 77,749 patients (71.6%) were prescribed combination antiemetic regimens, while 16,417 patients (15.1%) were prescribed a single antiemetic drug to treat HEC. Dual combination regimens were the most common, followed by triple combination therapy and monotherapy. 5‐HT_3_RAs and glucocorticoids were the two most frequently used classes, prescribed to patients receiving a single antiemetic drug (11.8% and 2.5%, respectively) and those on combination regimens (70.4% and 63.4%, respectively). The dual combination therapy commonly included a 5‐HT_3_RA with a glucocorticoid (31.7%), followed by a 5‐HT_3_RA with either metoclopramide or an NK1RA (4.9% and 1.2%, respectively). For triple combination therapy, a 5‐HT_3_RA and a glucocorticoid were often combined with metoclopramide or an NK1RA (14.3% and 5.3%, respectively). As for the quadruple combinations, only 103 patients (0.1%) received the recommended four‐drug regimen comprised of a 5‐HT_3_RA, a glucocorticoid, an NK1RA, and Olanzapine. Finally, overuse of antiemetic drugs from the same class was observed on a single prescription. As shown in Figures 3 and 4, 8.8% and 1.6% of patients were prescribed more than two 5‐HT_3_RAs and glucocorticoids in a combination regimen, respectively.

**TABLE 2:** The counts (*n*) and percentages (%) of main prescribed antiemetic patterns during the whole phases of cisplatin‐based chemotherapy‐induced nausea and vomiting from 2015 to 2020.

Prescription patterns	Year						Sum (*N* = 108,611)
	2015 (*N* = 16,108)	2016 (*N* = 17,244)	2017 (*N* = 18,728)	2018 (*N =* 19703)	2019 (*N* = 20,451)	2020 (*N* = 16,377)	
Monotherapy, *n* (%)	2455 (15.2)	2455 (14.2)	2496 (13.3)	3024 (15.3)	3214 (15.7)	2773 (16.9)	16,417 (15.1)
One 5‐HT_3_RA, *n* (%)	1980 (12.3)	1956 (11.3)	1972 (10.5)	2230 (11.3)	2516 (12.3)	2145 (13.1)	12,799 (11.8)
One GC, *n* (%)	385 (2.4)	390 (2.3)	420 (2.2)	569 (2.9)	503 (2.5)	492 (3.0)	2759 (2.5)
MCP, *n* (%)	87 (0.5)	104 (0.6)	85 (0.5)	194 (1.0)	146 (0.7)	98 (0.6)	714 (0.7)
Others, *n* (%)	3 (<0.1)	5 (<0.1)	19 (0.1)	31 (0.2)	49 (0.2)	38 (0.2)	145 (0.1)
Dual combinations, *n* (%)	6026 (37.4)	6312 (36.6)	7033 (37.6)	8973 (45.5)	8800 (43.0)	6279 (38.3)	43,423 (40.0)
One 5‐HT_3_RA + one GC, *n* (%)	4848 (30.1)	4935 (28.6)	5604 (29.9)	7475 (37.9)	7024 (34.3)	4490 (27.4)	34,376 (31.7)
One 5‐HT_3_RA + MCP, *n* (%)	800 (5.0)	832 (4.8)	962 (5.1)	919 (4.7)	959 (4.7)	895 (5.5)	5367 (4.9)
One 5‐HT_3_RA + one NK1RA, *n* (%)	31 (0.2)	101 (0.6)	143 (0.8)	167 (0.8)	356 (1.7)	490 (3.0)	1288 (1.2)
Two 5‐HT_3_RAs, *n* (%)	209 (1.3)	264 (1.5)	142 (0.8)	171 (0.9)	168 (0.8)	157 (1.0)	1111 (1.0)
One GC + MCP, *n* (%)	134 (0.8)	159 (0.9)	158 (0.8)	212 (1.1)	224 (1.1)	147 (0.9)	1034 (1.0)
Others, *n* (%)	4 (<0.1)	21 (0.1)	24 (0.1)	29 (0.1)	69 (0.1)	100 (0.3)	247 (0.2)
Triple combinations, *n* (%)	4049 (25.1)	4185 (24.3)	4580 (24.5)	4914 (24.9)	5619 (27.5)	4700 (28.7)	28,047 (25.8)
One GC + one 5‐HT_3_RA + MCP, *n* (%)	2935 (18.2)	2441 (14.2)	2628 (14.0)	2870 (14.6)	2751 (13.5)	1926 (11.8)	15,551 (14.3)
One GC + one 5‐HT_3_RA + one NK1RA, *n* (%)	69 (0.4)	490 (2.8)	712 (3.8)	680 (3.5)	1827 (8.9)	1987 (12.1)	5765 (5.3)
One GC + two 5‐HT_3_RAs, *n* (%)	737 (4.6)	833 (4.8)	861 (4.6)	943 (4.8)	653 (3.2)	317 (1.9)	4344 (4.0)
One 5‐HT_3_RA + Two GCs	206 (1.3)	244 (1.4)	148 (0.8)	140 (0.7)	75 (0.4)	45 (0.3)	858 (0.8)
One 5‐HT_3_RA + one GC + OLA, *n* (%)	15 (0.1)	17 (0.1)	93 (0.5)	147 (0.7)	124 (0.6)	74 (0.5)	470 (0.4)
One 5‐HT_3_RA + one NK1RA + MCP, *n* (%)	1 (<0.1)	14 (0.1)	41 (0.2)	69 (0.4)	80 (0.4)	135 (0.8)	340 (0.3)
Two 5‐HT_3_RAs + MCP, *n* (%)	74 (0.5)	105 (0.6)	64 (0.3)	22 (0.1)	40 (0.2)	28 (0.2)	333 (0.3)
Others, *n* (%)	12 (0.1)	41 (0.2)	33 (0.2)	43 (0.2)	69 (0.3)	188 (1.1)	386 (0.4)
Quadruple combinations, *n* (%)	602 (3.7)	1207 (7.0)	1278 (6.8)	809 (4.1)	1032 (5.0)	963 (5.9)	5891 (5.4)
Two 5‐HT_3_RAs + one GC + MCP, *n* (%)	425 (2.6)	565 (3.3)	498 (2.7)	282 (1.4)	121 (0.6)	95 (0.6)	1986 (1.8)
One NK1RA + one 5‐HT_3_RA + one GC + MCP, *n* (%)	25 (0.2)	255 (1.5)	325 (1.7)	215 (1.1)	451 (2.2)	547 (3.3)	1818 (1.7)
One NK1RA + two 5‐HT_3_RAs + one GC, *n* (%)	21 (0.1)	192 (1.1)	146 (0.8)	59 (0.3)	258 (1.3)	185 (1.1)	861 (0.8)
One 5‐HT_3_RA + one GC + MCP + OLA, *n* (%)	5 (<0.1)	28 (0.2)	87 (0.5)	92 (0.5)	30 (0.1)	42 (0.3)	284 (0.3)
One 5‐HT_3_RA+ Two GCs + MCP, *n* (%)	64 (0.4)	45 (0.3)	71 (0.4)	51 (0.3)	39 (0.2)	14 (0.1)	284 (0.3)
One NK1RA + one 5‐HT_3_RA + one GC + OLA, *n* (%)	0 (<0.1)	1 (<0.1)	12 (0.1)	17 (0.1)	39 (0.2)	34 (0.2)	103 (0.1)
Others, *n* (%)	62 (0.4)	121 (0.7)	139 (0.7)	93 (0.5)	94 (0.5)	46 (0.3)	555 (0.5)
Five combinations, *n* (%)	21 (0.1)	77 (0.4)	109 (0.6)	60 (0.3)	42 (0.2)	61 (0.4)	370 (0.3)
Six combinations, *n* (%)	0 (<0.1)	5 (<0.1)	4 (<0.1)	3 (<0.1)	2 (<0.1)	4 (<0.1)	18 (<0.1)

Abbreviations: GC, glucocorticoid; LOR, Lorazepam; MCP, Metoclopramide; NK1RA, neurokinin 1 receptor antagonist; OLA, olanzapine; 5‐HT_3_RA, 5‐hydroxytryptamine‐3 receptor antagonist.

### 3.5 Antiemetic guideline adherence

At the time the survey was performed, Chinese^10^ and international antiemetic guidelines^23^ recommended a triple‐drug regimen comprising an NK1RA, a 5HT_3_RA, and a glucocorticoid, with or without olanzapine, as the evidence‐based approach to treat HEC. AGA was defined as the use of combination regimens including triple prophylaxis (NK1RA, 5HT_3_RA, and glucocorticoid), with or without olanzapine, at the initiation of cisplatin‐based chemotherapy. The study found that 8699 patients (8.0%) received standard triplet therapy and 132 patients (0.1%) received olanzapine‐containing quadruple antiemetic regimen. The overall rate of AGA among patients treated with cisplatin‐based HEC regimens was low; thus, a comprehensive analysis of complete guideline adherence association with doses, frequencies, and durations of antiemetic drugs was not performed.

## 4 DISCUSSION

Due to the multifactorial causes of CINV, it is crucial to use a combination of antiemetic drugs from different classes to effectively prevent CINV caused by highly or moderately emetogenic chemotherapy, taking into account both the emetogenic potential of the chemotherapy regimen and the individual patient's risk factors.^3^ Throughout the study period, both Chinese and international antiemetic guidelines have recommended inclusion of an NK1RA and/or olanzapine in managing acute and delayed CINV associated with HEC. However, in China, NK1RAs were a new class of antiemetics, and olanzapine was used as an off‐label antiemetic drug. Little information is currently available regarding the integration of NK1RAs and/or olanzapine into HEC treatment in clinical practice.^19^ To our knowledge, this is the first study to apply real‐world data to comprehensively describe the utilization and trends of various antiemetic drugs, including NK1RAs and olanzapine, in the management of cisplatin‐based HEC‐induced nausea and vomiting in China.

Overall, our study has indicated that from 2015 to 2020, compliance with antiemetic guidelines for managing CINV associated with cisplatin‐based chemotherapy in China was markedly suboptimal. Despite consistent recommendations by antiemetic guidelines for the inclusion of a 5‐HT_3_RA, a glucocorticoid, and an NK1RA, optionally combined with olanzapine, to alleviate acute and delayed CINV in HEC, our findings suggest that nonadherence primarily resulted from the omission of one or more antiemetic drugs. Specifically, NK1RAs were prescribed in only 10.1% of cases, which significantly reduced overall guideline adherence. The limited use of NK1RAs may be due to their high cost and exclusion from medical insurance reimbursement during the study period. Moreover, a notable 34.0% of patients did not receive glucocorticoid treatment, potentially arising from oncologists' concerns regarding serious side effects and infection risks.^24^ Additionally, our study identified that only 1.1% of patients received olanzapine, an antipsychotic agent, despite its established efficacy in managing acute, delayed, and refractory CINV of various etiologies.^25^, ^26^ The off‐label prescription of olanzapine as an antiemetic may restrict its availability in hospitals that limit such use, along with persistent concerns about its safety profile, even though numerous studies have affirmed its tolerability.^27^ Our study also revealed an underrecognition of anticipatory CINV in the HEC setting, with the anxiolytic lorazepam, which is beneficial for this condition,^28^ being prescribed to only a small number of patients. Inadequate management of anticipatory CINV can significantly impact patient quality of life and adherence to chemotherapy treatments. Finally, our study indicated that 13.3% of cisplatin‐based treatments did not include any antiemetic drug, highlighting a potential underestimation of emetic risk by oncologists and a lack of familiarity with antiemetic guidelines.

Conversely, our study indicates overprescriptions of antiemetic drugs within the same class. 5‐HT_3_RAs were the most commonly prescribed, with 82.2% of patients receiving at least one such agent. Palonosetron, a second‐generation 5‐HT_3_RA, is considered more effective in preventing delayed CINV due to its extended half‐life and increased receptor affinity.^29^ Since 2016, palonosetron has surpassed tropisetron as the most frequently prescribed 5‐HT_3_RA, reflecting its superior efficacy in CINV control. However, there was an excessive use of 5‐HT_3_RAs, consistent with previous reports from China^19^ and Brazil.^30^ Among all cisplatin‐based treatments, 9329 patients (8.6%) were prescribed two 5‐HT_3_RAs, and 224 patients (0.2%) were prescribed three, which is not recommended by any guideline for acute and delayed CINV. Such overprescription may not provide additional symptom relief but could increase healthcare costs and potential risks. Additionally, our study observed an overprescription of glucocorticoids in 1727 patients (1.6%). Remarkably, metoclopramide was prescribed to 28,195 patients (26.0%) for CINV management. Historically, high‐dose metoclopramide was used for CINV; however, it carries the risk of causing tardive dyskinesia, a severe movement disorder lacking effective treatment.^31^ Due to newer, more effective medications and potentially serious adverse effects, metoclopramide is not recommended as a first‐line antiemetic drug for HEC and is usually used as an effective agent for breakthrough CINV according to guidelines. The widespread use of metoclopramide in the HEC setting by oncologists indicates that they were not aware of the exclusion of metoclopramide from guidelines for acute CINV induced by such treatments or the substitution of it with olanzapine.

Implementing effective measures is critical to promote the rational utilization of antiemetic drugs for CINV in China. First, medical insurance departments should prioritize the reimbursement of cost‐effective and evidence‐based antiemetic regimens, and insurance policies can incentive both healthcare providers and patients to adhere to best practices. Second, continuous education for healthcare professionals is essential and nationwide educational programs should be implemented to facilitate easy access to up‐to‐date guidelines and best practices for CINV management. Furthermore, a multidisciplinary team composed of oncologists, pharmacists, nurses, etc., should be established to collaboratively develop treatment plans and monitor medication effects to ensure the judicious use of antiemetic drugs. Moreover, developing clinical decision support tools based on established guidelines and recent research findings is crucial to assist oncologists in selecting and applying antiemetic drugs more effectively in clinical settings.

This study has significant strengths. It included a large sample size of updated antiemetic prescriptions, assessed over a long duration from a wide area, enabling the analysis of trends in antiemetic drug use and prescription habits. Consequently, the findings of our study may provide significant evidence and insights for improving CINV management in China and beyond.

However, this study also has limitations. One particular challenge is the inability to differentiate between acute and delayed phases of nausea and vomiting since the data were obtained solely from prescriptions rather than detailed patient medical records. In our analysis, we only considered the overall phase of each cycle (acute plus delayed phases), and the differences in prescription patterns between acute and delayed CINV remain unclear. Another limitation is that there may be bias in sampling. The majority of hospitals included in our study are tertiary hospitals located in large cities, which may not accurately represent the prescription patterns for Chinese patients receiving HEC in less economically developed and rural areas. A third limitation is that due to the limited number of patients receiving AGA therapy, we were unable to assess the impact of dose, frequency, and duration of antiemetic drugs on achieving complete symptom control. These topics are important and will be explored in further research.

In conclusion, this study highlights a significant discrepancy between guideline recommendations and the actual practice of antiemetic treatments in China. Only 8.1% of cisplatin‐based chemotherapy regimens adhered to the recommended antiemetic guideline combinations, which may further reduce when evaluating dose, frequency, and duration. The low adherence may be attributed to factors such as high cost of new antiemetics, off‐label use of certain antiemetic agents, underestimation of the emetogenic potential of specific chemotherapeutic drugs, and insufficient awareness of appropriate antiemetic therapy among oncologists. Therefore, policymakers and healthcare providers should pay more attention and take immediate measures to improve the management of CINV in cancer patients.

## AUTHOR CONTRIBUTIONS

**Xia Si:** Data curation (lead); formal analysis (lead); writing – original draft (lead). **Hongyan Zhang:** Data curation (supporting); formal analysis (supporting); software (lead). **Qingming Ding:** Data curation (supporting); methodology (lead). **Gang Liu:** Software (supporting); supervision (lead). **Lin Huang:** Conceptualization (equal); funding acquisition (lead); validation (equal); writing – review and editing (equal). **Xin Sun:** Conceptualization (equal); validation (equal); writing – review and editing (equal).

## FUNDING INFORMATION

This work was supported by grants from Peking University People's Hospital, China (2024‐Z‐13).

## CONFLICT OF INTEREST STATEMENT

The authors declare that the research was conducted in the absence of any commercial or financial relationships that could be construed as a potential conflict of interest.

## ETHICS STATEMENT AND CONSENT TO PARTICIPATE

The study was approved by the Ethics Committee of Peking University People's Hospital review board with the approval number of 2021PHB350‐001. The written informed consent from patients was waived because the data were analyzed anonymously.

## Data Availability

The data that support the conclusions of this article are available from the corresponding authors upon reasonable request.
